# Individual Acute Exposures to Low Concentrations of Cadmium, Chromium, Lead, and Nickel Affect Oxidative Stress and Pathological Markers in Fruit Bats

**DOI:** 10.1021/acsomega.5c00991

**Published:** 2025-05-08

**Authors:** Ana Luiza Fonseca Destro, Thaís Silva Alves, Fernanda Ribeiro Dias, Reggiani Vilela Gonçalves, Jerusa Maria de Oliveira, Leandro Licursi de Oliveira, Mariella Bontempo Freitas

**Affiliations:** a Department of Animal Biology, Federal University of Viçosa, Viçosa, MG 36570-900, Brazil; b Department of Structural Biology, Federal University of Triângulo Mineiro, Uberaba, MG 38025-350, Brazil; c Department of General Biology, Federal University of Viçosa, Viçosa, MG 36570-900, Brazil; d 28112 Federal University of Alagoas, Physics Institute, Maceió, AL 57072450, Brazil

## Abstract

We investigated the effects of heavy metals on fruit-eating bats. Artibeus lituratus, a species that is not endangered, received a 1.5 mg/kg intraperitoneal (ipi) injection of cadmium (Cd), chromium (Cr), lead (Ld), or nickel (Ni). After 96 h, Ni-exposed bats showed oxidative stress in the liver and testes; the kidneys showed increased vascular congestion. Pb-exposed bats showed lower glutathione *S*-transferase (GST) activity in all tested tissues and a decreased percentage of normal cells in the seminiferous tubules in the testes. Bats exposed to Cr showed lower GST activity in the kidneys and testes, higher leukocyte infiltrate in the liver, and higher vacuolization in the testes. Cd-exposed bats showed lower GST activity in all tissues, higher leucocyte infiltrate in the kidneys, and a lower percentage of normal cells in the testes. Necrotic and lipidic areas in the liver were observed in Pb-exposed and Ni-exposed bats. We propose the following toxicity order for fruit-eating bats: Ni > Pb > Cr = Cd.

## Graphical abstract

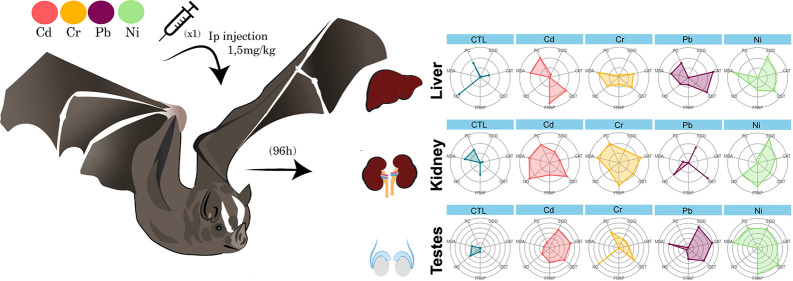

## Introduction

Heavy metals (HM) are often referred to in ecotoxicological studies as metals and metalloids with potential toxicity, usually associated with environmental pollution.^1^ Anthropogenic activities such as mining and industrialization have been a major concern as they might be involved in increased HM levels in the environment.^2^ As metals are not degraded in the environment, they may accumulate in living organisms and within food chains, processes known as bioaccumulation and biomagnification, respectively.^2^

Cadmium (Cd) is a nonessential heavy metal^3^ and could accumulate in organisms through food chains.^4^ In the male mammalian reproductive system. Low and prolonged Cd concentrations damaged different organs, such as the liver and kidneys.^5^ Chromium (Cr) is an essential nutrient for animals.^6^ Excessive Cr exposure was found to affect the mammalian reproductive system, inducing oxidative stress due to its strong oxidant capacity.^7^ Lead (Pb) is a nonessential element^3^ widely found in industrial products (batteries, paints, gasoline, pesticides, medicines, cosmetics, etc.) and is considered the most important toxic heavy element in the environment due to its abundant global distribution.^8^ Pb induces damage to the renal, reproductive, and nervous systems in mammals.^8^ Nickel (Ni) is an essential nutrient,^9^ and high Ni levels have been proven to disrupt the Hypothalamus-Pituitary-Gonad (HPG) axis and generate excessive reactive oxygen species (ROS)/reactive nitrogen species (RNS) production in testes.^10^

In the environment, variable concentrations of Cd, Cr, Pb, and Ni are often found, depending on the proximity of industrial activities and the natural composition of the soil, for instance, concentrations (mg/kg) up to 11.7 for Cd, 468 for Cr, 510 for Pb, and higher than 583 for Ni in the soil close to gold mining exploration have already been reported.^11^ These associated metals are often not bioavailable, although they can be released due to anoxic conditions associated with plant and animal activity.^12^ This environmental contamination may impact the local fauna in several ways, and factors like the chemical form of the metal in the environment, availability, concentration, exposure time, interaction with other metals in the environment, and the animal’s trophic level may influence the impact extent.^13^

Although the effects of HM, such as those cited above, have been described in several animal models, its effects on wild species are less understood. Bats are the only true flying mammals, with adaptations that include a high metabolic rate, high longevity, and a reduced inflammatory response (which may be associated with their ability to deal with viral infection with lower impact).^14^ Some of these specific traits could indicate that environmental pollutants may affect bats differently compared to other mammals. Despite this, the risk assessment of pollutants in bats is incipient and requires a specific approach for these animals.^15^ The great fruit-eating bat (Artibeus lituratus) is an abundant species in the neotropical region, where it contributes to reforestation, especially in heavily fragmented areas of the Atlantic Rain Forest.^16^

Although there are not many studies on this, scientific literature shows that bats can accumulate HM found in the environment. Concentrations (mg/kg) such as 5.8–7.32 of Pb, 5.7–10.9 of Cr, 3.6–4.05 of Cd, and 4.3–8.6 of Ni have already been found in insectivorous bats living in coal mining areas.^17^ However, there is a lack of information on the specific, isolated effect of the main environmentally available HM in bats exposed to each one of them, individually. Authors have reported the need for more studies on the effects of metals in bats, due to the difficulty in discussing and understanding in situ studies, which arises from the lack of laboratory studies conducted without the influence of other pollutants.^18–20^ In a region where mining operations and forest fragments are closely located, investigations of how HM pollution affects important aspects of local wildlife are needed to understand the extent of these impacts.

Here, we aimed to evaluate the toxicological effects of acute exposure to low concentrations of four HM (Cd, Cr, Pb, and Ni) on redox balance and histopathological parameters in physiologically relevant tissues of the great-fruit-eating bat. Our study was designed based on a previous study with mice,^21^ in order to make possible comparisons and assumptions regarding eventual differences between the two animal models.

## Materials and Methods

### Chemicals

The metals CdCl_2_ (cadmium chloride 99.9%), CrO_3_ (chromium VI trioxide ≥ 99%), Pb (CH_3_COO)^2^·3H_2_O (neutral lead acetate P.A. trihydrate), and Cl_2_Ni·6H_2_O (nickel­(II) chloride hexahydrate 99.9%) were obtained from Sigma-Aldrich (St. Louis, Missouri, US) and Merck (Darmstadt, Germany) and diluted in distilled water to obtain the target concentrations (1,5 mg/kg) of Cd, Cr, Pb, and Ni, according to other studies.^21^

### Animals

Adult male great fruit-eating bats (Artibeus lituratus (A. lituratus), *n* = 31, body weight = 73.62 ± 5.78 g) were captured using mist nets in a forest area from the Federal University of Viçosa (UFV) (20° 45′ S and 42° 52′ W), Viçosa, Minas Gerais, Brazil. All animals were under the same conditions, gender, and captured in the same location and during the same season, as described in other studies with bats.^22^ Animals were identified according to Díaz et al.,^23^ brought to the University, and kept in a half-wall screen-lined bat house located at the Museum of Zoology garden under trees of the Atlantic Forest. Bats were aleatorily assigned to individual enclosures (a total of 8 enclosures of 2 m^3^ size each), where they could fly freely inside the rooms, and kept under natural cycles of temperature, light, and humidity. All animals were submitted to a 4 day acclimation period before the exposure started. During this time, the animals were offered tropical fruits (Carica papaya, Musa sp., Psidium guajava, and Mangifera indica L.) and water *ad libitum*. All animal captures and procedures performed in this study were approved by the Brazilian Government (SISBIO 75064–1) and the Animal Ethics Committee (CEUA-UFV 26/2020).

### Experimental Design

Following the acclimation period, the animals were treated according to one of the following experimental groups: CTL) control: normal saline solution (NaCl 0.9%) (*n* = 6) and experimental groups that received 1.5 mg/kg of each metal in the following compounds: Cd) cadmium chloride (CdCl_2)_ (*n* = 6); Cr) chromium trioxide IV (CrO_3)_ (*n* = 6); Pb) lead acetate (Pb (CH_3_COO)^2^·3H_2_O) (*n* = 6) and Ni) nickel chloride (Cl_2_Ni.6H_2_O) (*n* = 7). We chose to run the CTL group with the saline solution due to the fact that metals were supplied in the form of salt in metal-exposed groups, so the saline solution would offer the CTL group similar conditions to the treated ones (Cupertino et al. and Albasher et al.).^24,25^ Exposure to saline or metals was performed through one intraperitoneal injection (ipi) of 0.7 mL of solution at 8:00 *Ante meridiem* (a.m.) on day 1 of exposure. The IPI route was chosen as it ensures that the total volume would be completely absorbed by the animal, avoiding consumption bias between metals. In addition, gavage in bats is often complicated and stressful due to the lack of proper tools, specifically designed for their unique anatomy, and IPI absorption is faster and more efficient than oral.^26^ Food (fruits) was offered each night at 6:00 *Post meridiem* (p.m.) (150–200 g each), and leftovers were weighed in the morning, according to De Oliveira et al., to make sure that all animals were fed to satisfaction.^21^ Water was available *ad libitum*. The metal concentrations we tested were chosen from previous similar experiments with adult male mice.^21^ Although these doses were already investigated in the testes of murine models, this study is the first to use bats as models, and the results will allow a comparison that will advance the understanding of how much the effects observed for murine models can be extrapolated to bats. This study is the first to use bats as models, and therefore, the effects of each metal and relevant concentration are unclear. The same concentrations for all metals were chosen to make comparisons among them possible. After 96 h of exposure, bats were euthanized through cervical dislocation followed by decapitation. The liver, kidney, and testes were rapidly removed under ice, divided into fragments, weighed, and portions assigned to the redox status determination were flash frozen in liquid nitrogen until storage at −80 °C. The other portion of these organs was assigned to histopathological analysis and fixed for subsequent investigation.

### Redox Status Determinations

#### Tissue Preparation

Samples were homogenized in 0.2 mol/L phosphate buffer and 1 mmol/L ethylenediaminetetraacetic acid (EDTA) (1,1 and 1.5:1, respectively), pH 7.4, using a tissue homogenizer (OMNI) (Kennesaw, USA). The homogenates were centrifuged at 15,000 g for 10 min at 4 °C prior to the analysis.

#### Assessment of Oxidative and Nitrosative Stress Markers

The homogenate supernatant was used for nitric oxide (NO), superoxide dismutase (SOD), catalase (CAT), glutathione *S*-transferase (GST), ferric reducing ability of plasma (FRAP), and malondialdehyde (MDA). An additional assay of protein total was done to standardize the results of CAT, SOD, and MDA. The remaining pellets were used for protein carbonyl assays. All samples were randomly assigned to blind analyses without sample identification until analysis of the results to avoid bias. All samples were run in duplicate using a spectrophotometer (UV-Mini 1240, Shimadzu, Japan) or a microplate reader (Thermo Scientific, Waltham, USA).

NO production was quantified by the standard Griess reaction. Briefly, 50 μL of supernatant described above were incubated with an equal volume of Griess reagent (1% sulfanilamide, 0.1% *N*-(1-naphthyl) ethylenediamine, and 2.5% phosphoric acid) at room temperature for 10 min.^27^ The absorbance was measured at 570 nm in a microplate reader. The conversion of absorbance into micromolar concentrations of NO was obtained from a sodium nitrite (0–100 μmol/L) standard curve and expressed as NO concentrations (μmol/L).

SOD activity was determined by the method based on the reduction of the superoxide (O^2–^) and hydrogen peroxide, thereby decreasing the auto-oxidation of pyrogallol.^28^ The reaction mixture contained 99 μL of potassium phosphate buffer (5 mmol/L, pH 8.0) and 30 μL of sample and was started by adding 15 μL of pyrogallol (100 μmol/L). The final reaction was measured by the absorbance at 570 nm. SOD activity was calculated as units per milligram of protein, with one U of SOD defined as the amount that inhibited the rate of pyrogallol autoxidation by 50%. Duplicates of standards and blank samples for SOD activity were prepared with and without pyrogallol, respectively.

CAT activity was determined by adapting the Hadwan and Abed^29^ method. Briefly, 5 μL samples were incubated with 100 μL of hydrogen peroxide (20 mmol/L), and 100 μL of sodium and potassium phosphate pH buffer (50 mmol/L, pH 7.0). After 3 min, the reaction was stopped with 150 μL of ammonium molybdate (32.4 mmol/L). A control test without hydrogen peroxide was used to exclude the interference of amino acids and proteins. The reading at 374 nm was performed in a spectrophotometer. To calculate the CAT activity, a standard curve was built with serial dilutions of hydrogen peroxide. The CAT activity was calculated as units (U) per milligram of protein, where one unit of CAT activity is defined as the amount of enzyme that decomposes one mmol of H2O2 for 1 min. CAT activity was expressed in CAT KU/milligrams of protein, where KU is 1000U of CAT activity.

GST activity was measured using the method of Habig et al., 1974.^30^ Briefly, 1 mmol/L glutathione-conjugated 1-chloro-2,4-dinitrochlorobenzene (CDNB) was added to the buffer containing 1 mmol/L GSH and to an aliquot (10 μL) of the homogenate supernatant. Upon the addition of CDNB, the alteration was monitored through the absorbance at 340 nm for 60 s. The molar extinction coefficient used for CDNB was ε340 = 9.6 mmol/L × cm. One unit of GST activity was defined as the amount of enzyme that catalyzed the formation of one μmol of product/min/mL. GST activity was expressed in μmol/min/g.

The total antioxidant capacity was estimated according to the ferric reducing antioxidant power (FRAP), a method described by Benzie and Strain using TPTZ (2,4,6-Tris­(2-pyridyl)-*s*-triazine) as a substrate.^31^ The method is based on the reduction of a ferric 2,4,6-tripyridyl-*s*-triazine complex (Fe^3+^-TPTZ) to the ferrous form (Fe^2+^-TPTZ). Samples (10 μL) were added as FRAP solution (190 μL) of 25 mL of acetate buffer (300 mmol/L, pH 3.6), 2.5 mL of TPTZ reagent (10 mmol/L), and 2.5 FeCl_3_·6H_2_O solution (20 mmol/L), and the increase in absorbance at 593 nm was measured. The reducing Fe^3+^-TPTZ reagent by antioxidants was determined by using the standard curve of serial dilutions of FeSO_4_·7H_2_O starting with 1 mmol/L. The results were expressed as the FRAP value.

Malondialdehyde (MDA) is the major product of lipid peroxidation. MDA was measured according to Buege and Aust.^32^ Briefly, 0.2 mL of the tissue supernatant was homogenized in a solution (0.4 mL) of trichloroacetic acid (15%)/thiobarbituric acid (0.375%)/hydrochloric acid (0.6%). The total reaction mixture was kept in a boiling water bath for 40 min. After cooling on ice, butyl alcohol (0.6 mL) was added, and then the solution was vortexed for 2 min and centrifuged for 10 min at 9000*g*. The supernatant was used to measure the absorbance at 535 nm. The concentration of MDA was determined using the standard curve of known concentrations of 1,1,3,3-tetramethoxypropane (TMPO). The results were expressed as μmol/L per mg protein.

Protein carbonyl (PC) content was measured using 2,4-dinitrophenylhydrazine (DNPH), according to Levine et al.^33^ The homogenate pellet was added to 0.5 mL of DNPH solution (10 mmol/L) diluted in hydrochloric acid (7%), vortexed, and kept at room temperature in the dark, shaking periodically for 30 min. Then, 0.5 mL of ice-cold 10% trichloroacetic acid (TCA) was added to each tube, which was centrifuged (5000*g* for 10 min at 4 °C), and the supernatant was discarded. The precipitate was washed three times with 1 mL of ethyl acetate and ethanol (1:1 v/v). Finally, 1 mL of sodium dodecyl sulfate (SDS) 6% was added, the tubes were vortexed, and the supernatant was measured through absorbance at 370 nm. The results were expressed as nanomoles per milligram of protein based on the molar extinction coefficient of ε370 = 22 mmol/L × cm.

Total protein was determined according to Lowry et al.^34^ using bovine serum albumin (BSA) as a standard. Total protein concentrations were used to standardize the CAT, SOD, MDA, and PC results.

#### Integrated Biomarker Response (IBR)

To integrate the results from different biomarkers and understand the global response, we calculated the integrated biomarker response (IBR), following the method developed by Devin et al.,^35^ using the program CALIBRI (Calculate IBR Interface). The mean (*m*) and standard deviation (*s*) of a given biomarker were measured, and the group mean (*X*) is the mean value for the biomarker for a group. After that, we calculated a standardization for each group to obtain *Y*:

Y=(X−m)/s

Then, instead of transforming each biphasic biomarker into two variables with positive and negative values relating to biomarker inhibition and activation, we used the square form control *Y* score:

$$Z=(Y_{\mathrm{control}}-Y{)}^{2}$$

The score results (*S*) are

*S* = *Z* + |Min|, where *S* ⩾ 0 and |Min| are the lowest absolute values of *Z*.

Star plots were then used to display and calculate the integrated biomarker response (IBR), where the IBR is the star plot’s total area. As the results for each organ are a set of biomarkers, the ray coordinate of the star chart represents the score of a given biomarker in each organ. To avoid the strong dependencies on the biomarker arrangement along the star plot, the program uses a permutation procedure leading to (*k* – 1)!/2 possible values, where *k* is the number of biomarkers. The final IBR score is the mean of all IBR values corresponding to every possible order of biomarkers along the star plot.

### Histological Analysis

The left testes, left kidney, and a portion of the liver were fixed in 4% paraformaldehyde for 24 h and transferred to 70% ethanol. Tissue fragments were dehydrated in a growing series of ethanol and embedded in a glycol methacrylate (Historesin, Leica, Germany). Semiserial sections (3 μm) (12/animal) were made using a rotary microtome (RM 2255, Leica, Germany), with a minimum of 40 μm between sections, and stained with toluidine blue/sodium borate (testes) or hematoxylin/eosin (HE) (liver). Morphometry and stereology were performed using 10 digital images per animal, captured with a light microscope (Olympus BX-60, Tokyo, Japan) connected to a digital camera (Olympus QColor-3, Tokyo, Japan).

Liver images were morphometrically analyzed using grids with 266 intersections (Image Pro Plus 4.5 Software, Media Cybernetics, Silver Spring, Silver Spring, CA, USA). For stereological analysis, a test system of 266 points was used in a standard test area. In sections stained with HE, points were recorded in liver components (cytoplasm and nucleus of hepatocytes, blood vessels), inflammatory infiltrate, and congestion, with a total of 2660 points/animals.

In the kidneys, images obtained were analyzed morphometrically by counting the intersection of points on the glomeruli, renal tubules, and blood vessels, totaling 5320 points per animal. In addition, the radius and glomerular area, and the number of glomeruli present in each image were measured. For histopathological analysis, counts of leukocyte infiltration, vascular congestion, and leukocyte marginalization were performed, with 2660 points per animal. Analyses were performed by grids with 266 intersections of Image Pro Plus 4.5 Software (Media Cybernetics, Silver Spring, USA).

In the testis, the mean tubular diameter was obtained after measuring 30 random circular seminiferous tubule cross sections from each animal, regardless of the tubular stage (200× magnification). The seminiferous epithelium height was measured in the same tubular sections in which the tubule diameter was obtained (as the mean of the two Institutes of Health). Histopathological evaluation scores were used adapted from Johnsen, 1970 ^36^ to classify degenerative damage into normal – intact seminiferous tubules with germ cells in their normal places and few vacuoles; mild – vacuoles at the base or apex of the epithelium; moderate – vacuoles at the base and apex; or severe – tubules with only basal cells or only Sertoli cells.

### Statistical Analysis

Data distribution was determined by the Shapiro–Wilk test using the program GraphPad Prism 6.0 (San Diego, CA, USA). All data were submitted to a unifactorial one-way analysis of variance (ANOVA), followed by the Tukey post hoc test for multiple comparisons. When the distribution was not considered normal, the data were submitted to the Kruskal–Wallis test followed by Dunn’s test. Results are expressed as the mean and standard error of the mean (mean ± SEM). Statistical significance was established at *p* < 0.05.

## Results

### Redox Status

In the liver, SOD activity increased (χ^2^=16.99; *p* = 0.002) in Pb-exposed groups compared to CTL. GST activity decreased (*F*_(4,24)_ = 5.63; *p* = 0.002) in Cd (*p* = 0.008), Pb (*p* = 0.003), and Ni (*p* = 0.005) exposed groups compared to CTL. MDA concentration increased (*F*_(4,25)_ = 2.913; *p* = 0.042) only in Ni-exposed groups (*p* = 0.036). NO, CAT, FRAP, and PC did not differ among the groups (Table 1).

**Table 1:** Levels of Nitric Oxide Production (NO) (μmol/L), Activity of Superoxide Dismutase (SOD) (U/mg Protein), Catalase (CAT) (KU/mg Protein), Glutathione *S*-transferase (GST) (μmol/min/g), Total Antioxidant Capacity (FRAP) (μM), Malondialdehyde (MDA) (μmol/mg Protein), and Carbonylated Protein (PC) (nmol/mg Protein) in Tissues from A. lituratus Following 96 h of Exposure to Heavy Metals^a^

		NO	SOD	CAT	GST	FRAP	MDA	PC
liver	CTL	47.33 ± 8.60	0.92 ± 0.12	235.00 ± 25.68	24.42 ± 2.90	675.40 ± 81.12	0.17 ± 0.03	2.16 ± 0.50
	Cd	28.42 ± 10.500	1.02 ± 0.07	267.60 ± 40.73	11.19 ± 3.37*	492.70 ± 15.79	0.29 ± 0.05	2.47 ± 0.38
	Cr	38.42 ± 4.41	1.01 ± 0.09	211.4 ± 19.98	14.18 ± 1.01	622.40 ± 58.46	0.31 ± 0.02	1.42 ± 0.05
	Pb	35.76 ± 2.61	0.89 ± 0.07	173.20 ± 35.9	9.72 ± 1.99*	620.70 ± 58.46	0.28 ± 0.03	2.18 ± 0.72
	Ni	35.47 ± 4.07	2.40 ± 0.37*	198.40 ± 29.71	10.89 ± 1.99*	580.60 ± 28.09	0.33 ± 0.03*	1.34 ± 0.27
kidneys	CTL	4.39 ± 0.93	2.45 ± 0.3	3.02 ± 0.27	6.81 ± 0.27	432.40 ± 46.88	0.61 ± 0.05	21.82 ± 2.67
	Cd	12.68 ± 1.23*	2.19 ± 0.15	2.65 ± 0.25	8.78 ± 0.21*	473.40 ± 43.22	0.64 ± 0.04	25.79 ± 3.17
	Cr	8.37 ± 0.77	2.16 ± 0.16	2.09 ± 0.22	8.41 ± 0.35*	324.10 ± 37.98	0.64 ± 0.06	26.93 ± 4.22
	Pb	10.22 ± 0.74*	2.08 ± 0.05	3.13 ± 0.21	9.01 ± 0.34*	521.30 ± 33.07	0.56 ± 0.04	11.08 ± 1.18
	Ni	10.46 ± 1.47*	1.87 ± 0.11	2.30 ± 0.16	8.25 ± 0.36*	318.90 ± 37.83	0.54 ± 0.04	13.52 ± 0.76
testis	CTL	9.55 ± 1.04	4.85 ± 0.21	315.20 ± 18.41	2.78 ± 0.27	325.40 ± 20.06	2.34 ± 0.48	13.11 ± 1.10
	Cd	8.70 ± 1.09	7.54 ± 0.77	428.80 ± 50.20	1.67 ± 0.25*	252.90 ± 27.36	2.16 ± 0.73	16.44 ± 3.86
	Cr	12.86 ± 1.96	6.40 ± 0.90	368.60 ± 55.45	1.64 ± 0.14*	346.10 ± 19.79	1.69 ± 0.62	22.32 ± 3.86
	Pb	6.90 ± 1.06	7.87 ± 0.24	442.70 ± 66.48	1.62 ± 0.14*	286.40 ± 41.25	3.22 ± 1.19	13.54 ± 1.24
	Ni	6.28 ± 1.03	7.66 ± 0.84	419.30 ± 94.21	1.22 ± 0.14*	207.80 ± 47.54	3.61 ± 1.48	24.96 ± 2.93*

a CTL: control, Cd: cadmium, Cr: chromium, Pb: lead, Ni: nickel. Asterisk means statistical differences among groups (*P* ≤ 0.05). Data are shown as mean ± SEM.

In the kidney, NO content values increased (*F*_(4,24)_ = 6.841; *p* = 0.0008) in the Cd (*p* = 0.0004), Pb (*p* = 0.0144), and Ni (*p* = 0.0076) exposed groups compared to CTL. In GST activity, all exposed groups Cd (*p* = 0.0032), Cr (*p* = 0.0140), Pb (*p* = 0.0010), and Ni (*p* = 0.0317) increased (*F*_(4,22)_ = 6.668; *p* = 0.0011) compared to CTL. SOD, CAT, FRAP, and PC did not differ among the groups.

In the testes, GST activity decreased (*F*_(4,24)_ = 7.959; *p* < 0.001) in Cd (*p* = 0.007), Cr (*p* = 0.006), Pb (*p* = 0.007), and Ni (*p* < 0.001) exposed groups compared to CTL. PC concentration increased (*F*_(4,25)_ = 3.928; *p* = 0.013) in the Ni (*p* = 0.029) exposed group, and the other parameters did not differ among groups.

### Integrated Biomarker Response (IBR)

The IBR star plots of the liver, kidney, and testes are shown (Figure S1).

All groups exposed to HMs in the liver and testes had an increase in global damage. In the kidney, the groups exposed to Cd, Cr, and Ni had global damage increased when compared to that of the CTL group.

### Histological Analyses

Liver histological analyses did not show any differences in the percentage of liver nucleus, cytoplasm, blood vessels, and vascular congestion compared with the control. However, we found an increase (*F*_(4.17)_ = 3.635, *p* = 0.026) in leukocyte infiltrates in Cr (*p* = 0.018), fatty foci were observed in Pb, and areas of necrosis were observed in Pb- and Ni-exposed groups compared to the control (Table 2, Figure 1).

**Table 2:** Liver Histological Parameters from A. lituratus Following 96 h of Exposure to Heavy Metals^a^

treatments					
	CTL	Cd	Cr	Pb	Ni
nucleus (%)	8.16 ± 0.82	9.62 ± 0.55	9.94 ± 0.80	9.01 ± 3.81	9.96 ± 0.90
cytoplasm (%)	75.05 ± 1.34	73.01 ± 2.11	69.59 ± 1.58	76.90 ± 2.28	67.63 ± 2.37
blood vessels (%)	15.53 ± 0.18	18.76 ± 2.47	14.32 ± 0.04	13.61 ± 1.30	20.29 ± 1.07
vascular congestion (%)	8.47 ± 1.21	5.94 ± 1.16	8.61 ± 0.73	12.35 ± 1.21	7.91 ± 0.75
leukocyte infiltrate (%)	0.00 ± 0.00	0.45 ± 0.18	1.38 ± 0.48*	0.92 ± 0.30	0.54 ± 0.17

a CTL: control, Cd: cadmium, Cr: chromium, Pb: lead, Ni: nickel. Asterisk means statistical differences among groups (*P* ≤ 0.05). Data are shown as mean ± SEM.

**Figure 1:**
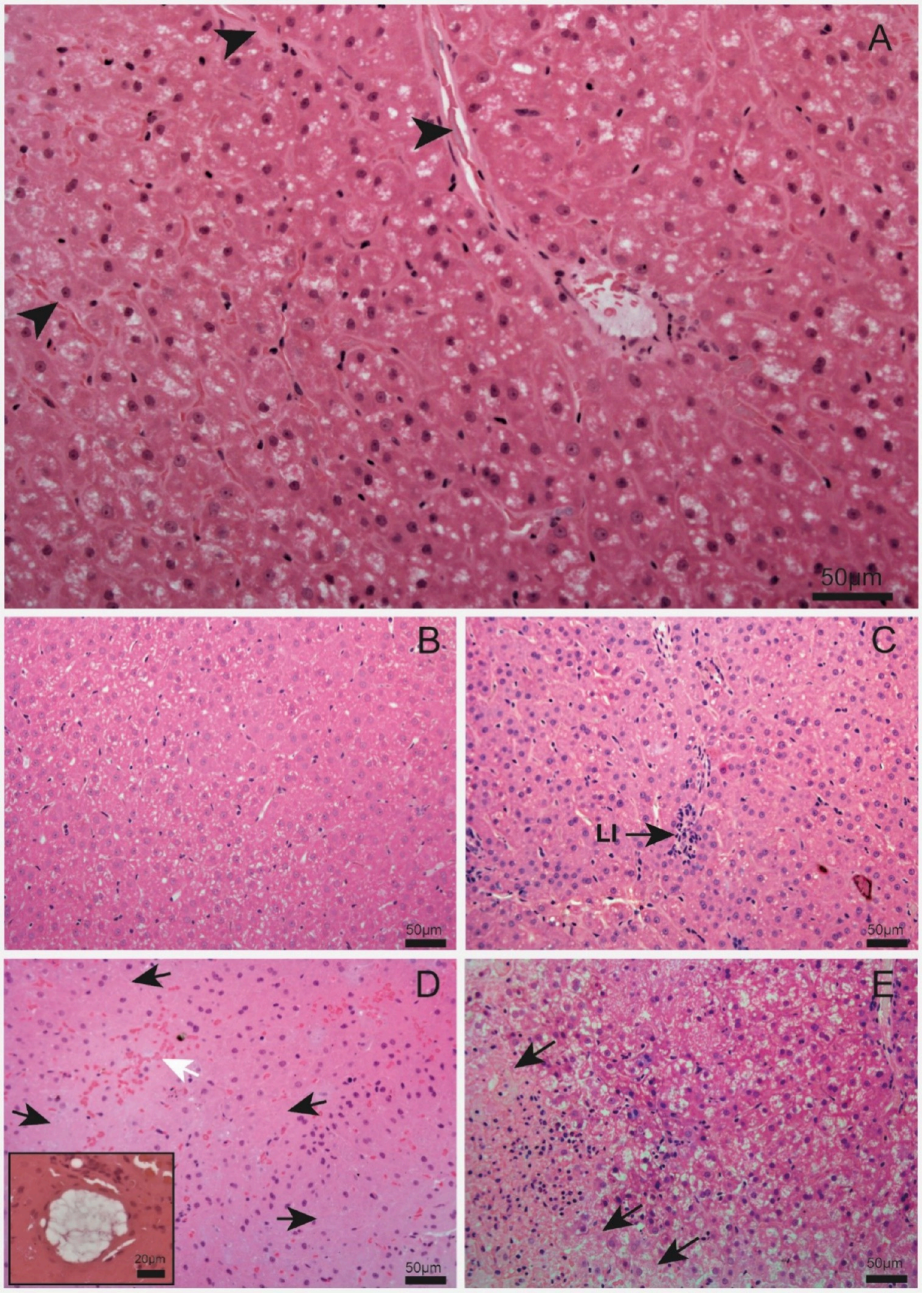
(A–E) Liver sections from A. lituratus from the following treatment groups: (A) CTL: control; (B) Cd: cadmium; (C) Cr: chromium; (D) Pb: lead; (E) Ni: nickel (HE staining, 20× objective lens). The highlighted image in part (D) indicates a fatty focci (40× objective lens). Black arrowhead: blood vessels; LI: leukocyte infiltrate; black arrow: necrotic area; white arrow: hemorrhage.

Histomorphometry parameters showed a decrease in capsular space (*X* ^2^ = 17.95, *p* = 0.0013) in Cd (*p* = 0.0073) and Ni (*p* = 0.0019), an increase in the tubular epithelium (*F*_(4.24)_ = 5.688, *p* = 0.0023) in Pb (*p* = 0.0171) and an increase in blood vessels (*F*_(4.24)_ = 15.17, *p* < 0.0001) in Cd (*p* = 0.0005), Cr (*p* < 0.0001), and Pb (*p* < 0.0001) and glomerulus radius (*F*_(4.25)_ = 10.15, *p* < 0.0001) in all groups (Cd: *p* = 0.0076, Cr: *p* = 0.0261, Pb: *p* = 0.0004, Ni: *p* < 0.0001), and also in the glomerulus area (*F*_(4.24)_ = 6.181, *p* = 0.0014) in Pb (*p* = 0.0294) and Ni (*p* = 0.0008). In the histopathological parameters, there was an increase (*F*_(4.25)_ = 5.398, *p* = 0.0028) in vascular congestion in Ni (*p* = 0.0019) and an increase (*F*_(4.24)_ = 3.466, *p* = 0.0227) in leukocyte infiltrate in Cd (*p* = 0.0106) (Table 3; Figure 2).

**Table 3:** Kidney Histological Parameters from A. lituratus Following 96 h of Exposure to Heavy Metals^a^

treatments					
	CTL	Cd	Cr	Pb	Ni
glomerulus (%)	2.95 ± 0.17	3.62 ± 0.11	4.45 ± 0.39	4.01 ± 0.52	4.31 ± 0.50
capsular space (%)	0.95 ± 0.20	0.16 ± 0.05*	0.35 ± 0.05	0.28 ± 0.08	0.13 ± 0.03*
tubular epithelium (%)	84.74 ± 0.29	86.24 ± 0.21	85.37 ± 0.92	87.72 ± 0.56*	84.51 ± 0.36
tubular lumen (%)	3.83 ± 0.36	4.09 ± 0.15	3.75 ± 0.15	3.53 ± 0.26	4.37 ± 0.17
blood vessels (%)	7.81 ± 0.47	5.89 ± 0.12*	5.43 ± 0.20*	5.10 ± 0.12*	6.71 ± 0.30
glomerulus radius (μm)	0.28 ± 0.01	0.35 ± 0.01*	0.34 ± 0.02*	0.37 ± 0.01*	0.39 ± 0.01*
glomerulus área (μm^2^)	27.48 ± 1.37	33.76 ± 1.89	30.99 ± 1.53	35.26 ± 1.51*	38.9 ± 2.24*
vascular congestion (%)	5.93 × 10^–3^ ± 5.51 × 10^–4^	1,07 × 10^–2^ ± 1.15 × 10^–3^	1.48 × 10^–2^ ± 2.58 × 10^–3^	9.82 × 10^–3^ ± 2.28 × 10^–3^	1.96 × 10^–2^ ±&nbsp;3.41 × 10^–3^*
leukocyte infiltrate (%)	4.90 × 10^–3^ ± 1.40 × 10^–3^	1.36 × 10^–2^ ± 1.75 × 10^–3^*	7.66 × 10^–3^ ± 1.66 × 10^–3^	9.58 × 10^–3^ ± 2.03 × 10^–3^	9.18 × 10^–3^ ± 1.64 × 10^–3^

a CTL: control, Cd: cadmium, Cr: chromium, Pb: lead, Ni: nickel. Asterisk means statistical differences among groups (*P* ≤ 0.05). Data are shown as mean ± SEM.

**Figure 2:**
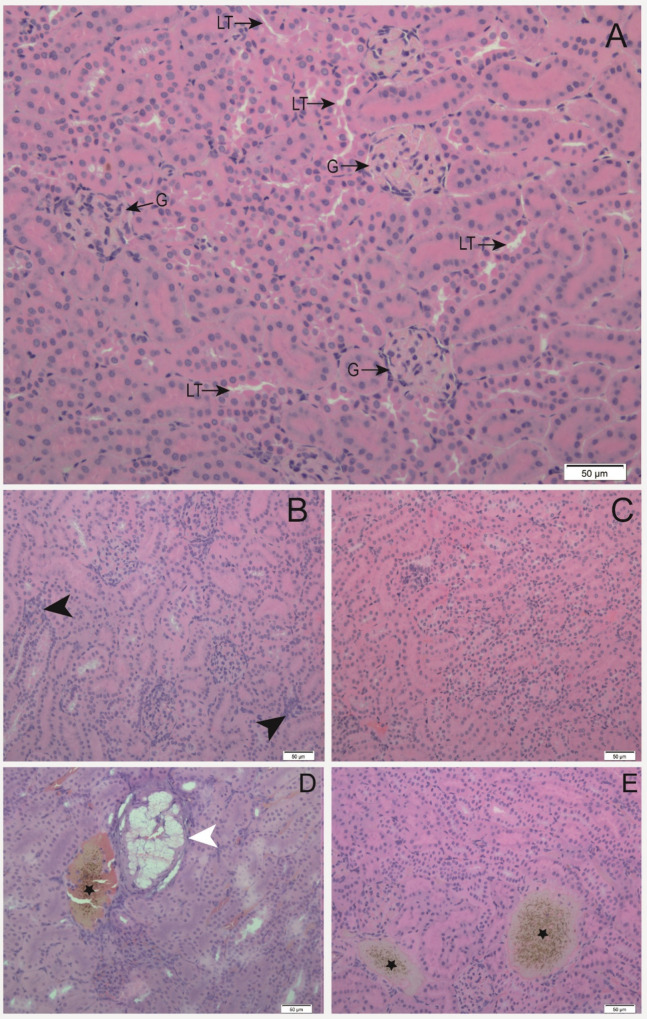
Kidney sections, the proportion of histopathological components in the kidney of A. lituratus from groups after 96 h to (A) CTL: control, (B) Cd: cadmium (C) Cr: chromium (D) Pb: lead (E) Ni: nickel. G: glomerulus; LT: tubular lumen; black arrowhead: leukocyte infiltrate; white arrowhead: fatty focci; star: vascular congestion (Hematoxylin and Eosin staining, 20× objective lens).

In the testes, we found an increase in luminal diameter (*F*_(4.18)_ = 4.388, *p* = 0.0119) in Cr-exposed bats (*p* = 0.0171) compared to the control. We also found a decrease (*F*_(4.20)_ = 12.16, *p* < 0.0001) in normal cells in all groups and an increase in vacuoles at the base (*F*_(4.19)_ = 7.646, *p* = 0.0008) and at the apex and base (*F*_(4.20)_ = 3.675, *p* = 0.0212) in Cr-exposed bats compared to control (*p* = 0011 and *p* = 0.0150, respectively). As for the sum of mild histopathology, there was an increase (*F*_(4.19)_ = 4.556, *p* = 0.0095) in Cr (*p* = 0.0057) and Ni (*p* = 0.0448) exposed groups bats and an increase in moderate histopathologies in Cr animals (*p* = 0.0147) compared to control (Table 4, Figure 3).

**Table 4:** Testes Histological Parameters from A. lituratus Following 96 h of Exposure to Heavy Metals^a^

treatments					
	CTL	Cd	Cr	Pb	Ni
tubular diameter (μm)	119.40 ± 6.90	147.90 ± 4.36	139.40 ± 15.67	118.90 ± 8.72	120.30 ± 0.81
luminal diameter (μm)	20.46 ± 2.90	22.42 ± 3.39	35.35 ± 4.08*	22.00 ± 1.39	29.58 ± 3.10
epithelium height (μm)	49.5 ± 2.35	59.68 ± 4.27	52.00 ± 6.17	49.16 ± 0.85	44.66 ± 3.09
normal cells (%)	90.06 ± 1.27	48.40 ± 7.42*	32.20 ± 5.21*	29.40 ± 11.19*	36.10 ± 6.95*
vacuole at the apex (%)	9.74 ± 1.22	25.10 ± 6.24	24.30 ± 6.46	29.00 ± 6.72	28.80 ± 8.33
vacuole at the base (%)	0.00 ± 0.00	6.30 ± 1.94	24.30 ± 6.46*	2.30 ± 1.28	10.50 ± 2.80
vacuole at the apex and base (%)	0.00 ± 0.00	15.30 ± 5.47	31.80 ± 11.01*	7.80 ± 2.19	20.30 ± 6.61
epithelial desquamation (%)	0.00 ± 0.00	1.20 ± 0.87	0.00 ± 0.00	1.63 ± 0.94	0.60 ± 0.37
seminiferous tubules with only basal cells (%)	0.00 ± 0.00	3.70 ± 2.24	2.40 ± 0.89	18.60 ± 11.12	3.10 ± 2.04
seminiferous tubules with only Sertoli cells (%)	0.00 ± 0.00	0.00 ± 0.00	0.00 ± 0.00	6.80 ± 4.45	0.60 ± 0.40

a CTL: control. Cd: cadmium. Cr: chromium. Pb: lead. Ni: nickel. Asterisk means statistical differences relative to CTL groups (*P* ≤ 0.05). Data are shown as mean ± SEM.

**Figure 3:**
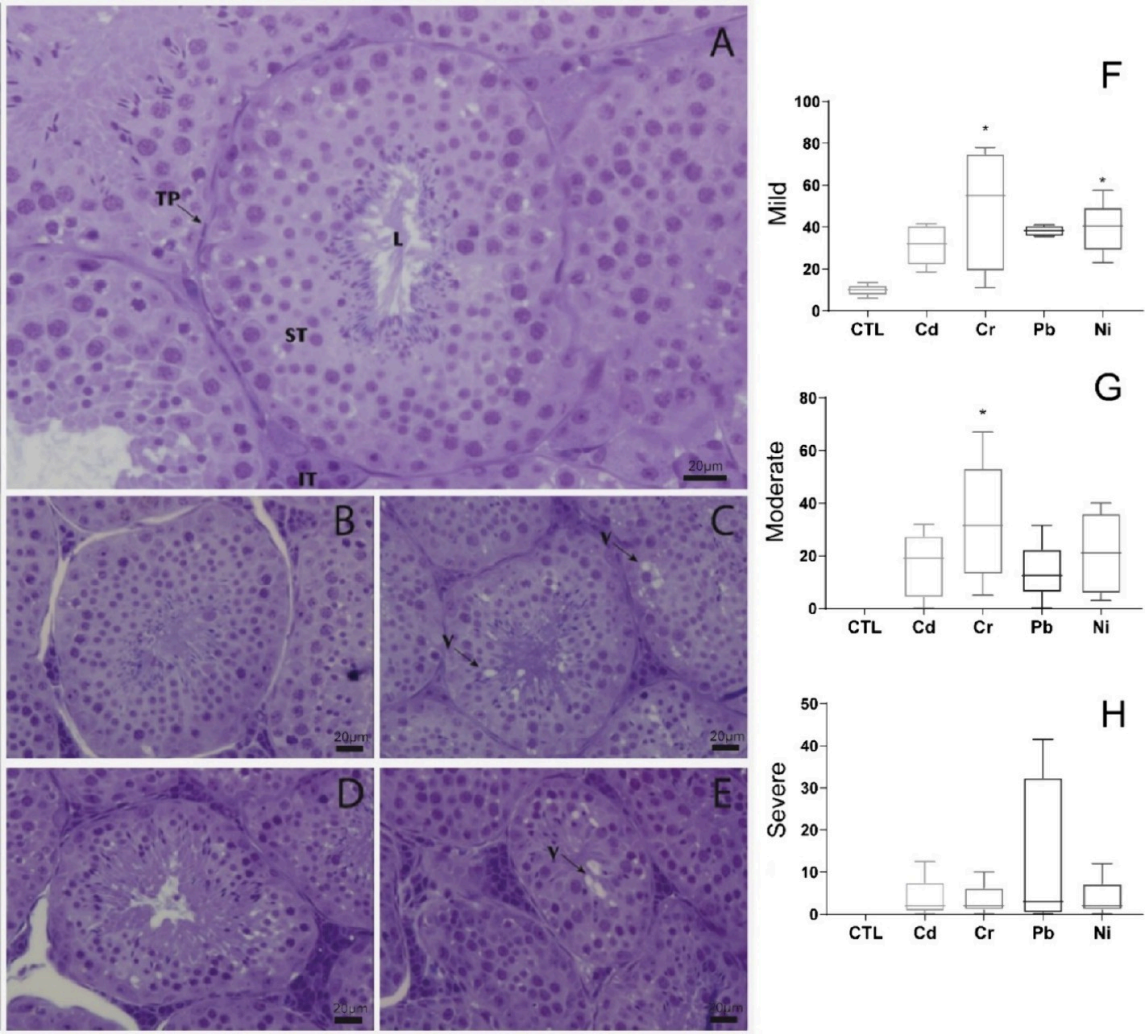
(A–E) Testis sections from A. lituratus from the following treatment groups: (A) CTL: control, (B) Cd: cadmium, (C) Cr: chromium, (D) Pb: lead, and (E) Ni: nickel (Toluidine Blue staining, 40× objective lens). TP: tunica propria; L: lumen; ST: seminiferous tubules; IT: intertubule; V: vacuole regions. (F–H) Johnsen’s (1970) histopathological evaluation score for degenerative damage in A. lituratus. *Asterisk means statistical differences relative to CTL groups (*P* ≤ 0.05). The data shown represent the median and interquartile range.

## Discussion

This is the first study to evaluate the isolated effect of heavy metals on wild bats. The concentrations analyzed in this study are lower than the concentrations found in insectivorous bats living in coal mining areas^17^ and lower than those found in tropical basin sediments in Brazil,^37^ so they are considered low concentrations.

### Liver Alterations

The analysis of IBR in the liver demonstrates that exposure to all metals harms the organ, even in low concentrations. Environmental studies showed that insectivorous bats living in coal mining areas can bioaccumulate higher metal concentrations in the liver than those analyzed in our study, such as 5.8–7.32 of Pb, 5.7–10.9 of Cr, 3.6–4.05 of Cd, and 4.3–8.6 of Ni (mg/kg).^17^ In the liver, antioxidant enzymes were altered by Cd, Ni, and Pb, which affected SOD and GST differently (Ni increased the SOD activity, while Cd, Pb, and Ni decreased the GST activity). Here, Ni exposure increased SOD activity, which may be associated with hydrogen peroxide (H_2_O_2_) overproduction.^38^ In our study, there was no increase in the CAT activity following Ni exposure. GST activity decreased, and that may indicate this enzyme could no longer protect the cells from lipid peroxidation,^39^ corroborating the increased MDA observed in this group. Decreasing GST activity following Cd, Ni, and Pb exposure might be explained by this metal’s ability to bind to thiol (−SH) groups on proteins and make them inactive, thus decreasing the activity of thiol-containing antioxidants, such as reduced glutathione (GSH),^40^ we suggest that this decreased activity of GSH also results in GST activity decreases since GST catalyzes the conjugation of GSH to toxics electrophiles.^41^ Corroborating this, a decrease in GSH activity and sulfhydryl content was observed in lungs from Artibeus lituratus collected in a mining area.^42^

Although morphometric analysis showed alteration, we observed increased leukocyte infiltrate in the liver of Cr-exposed bats, indicating inflammation. In murine models, exposure to higher Cr concentrations for 1–7 days induced pro-inflammatory cytokines and chemokines the release, which may result in the recruitment of inflammatory cells.^43^ In our study, histological hepatic images showed necrotic areas and hemorrhage in Ni-exposed animals and fat deposits and necrosis in the Pb-exposed group. Lipid degeneration in Pb-exposed rats has been previously documented when animals were treated through oral ingestion at a concentration of 20 mg/kg for 4 weeks, higher than that tested in this study.^44^

### Kidney Alterations

The global analysis of the kidney showed that exposure to metals (mainly Cr, Cd, and Ni) was toxic. The antioxidant enzymes were influenced by Cd, Cr, Ni, and Pb in different ways. NO increased in all of the exposed groups. NO has several functions in the kidneys as the regulation of renal hemodynamics and tubuloglomerular feedback.^45^ NO produced by the macula densa in the kidneys inhibits tubular sodium reuptake, resulting in increased urinary excretion and water and solutes.^46^ This can contribute to the excretion of toxic compounds. Levels of the CAT and SOD activity were not observed, except in GST for all groups. In the kidney, GST activity increased, unlike in other tissues where GST activity decreased, which may indicate an effort by the kidney to detoxify heavy metals, a defense mechanism since GST is a nonenzymatic detoxification defense.

In kidney histology, we found damage, although it does not indicate damage to MDA and PC. One possible reason for the decrease in Capsular space is a response to a decrease in blood pressure, indicated by increased vascular congestion. The narrowing of blood vessels found in all groups except Ni may indicate an attempt to avoid contamination of the kidneys by metals. The glomeruli enlargement in the same groups may represent a response to this blood deprivation. The increase in the level of leukocyte infiltrate observed in the group exposed to Cd demonstrates that this metal can induce a faster response to inflammation in the kidneys. The kidneys of bats collected in an area of pollution showed an increase in infiltrates and necrosis, compared to the control area.^47^ We did not find tissue necrosis, but some bats from the Pb group had large foci of fat, as we found in liver histology, which is a severe pathology. The fatty focci presents morphological characteristics similar to those of a lipoma; however, complementary analyses, especially to demonstrate tissue encapsulation and thus confirm our hypothesis, would be necessary.

### Testes Alterations

The IBR index for testes showed that all HMs presented a global increase in damage levels, highlighting Ni that doubled compared to the other metals. Regarding the antioxidant defense, only GST was affected by HM exposure in the testes, decreasing in all exposed groups. GSTs are a family of antioxidant isoenzymes that participate in the cellular detoxification of several xenobiotics, and the inhibitory effects of metals on GST activity may be harmful to the cell.^48^ Several studies showed decreased GST activities following HM exposure in testes of mammals, such as decreased GST after 0.025 mg/kg of Pb and Cd (ipi) for 15 days in rats.^49^ Differently from our study, chronic exposure to the same concentrations and the same metals did not show alterations in GST in testes, but Cr and Pb had alterations in SOD.^21^ Also, this same study found alterations in PC for Ni exposure, as ours,^21^ reinforcing our results that Ni is one of the most harmful metals, principally to the testis. Ni exposure also showed higher mild histopathologies. Cr exposure induced moderate histopathologies in the testes, increasing lumen diameter, indicative of tubular damage. Furthermore, all metals induced a decrease in normal tubular cells. That can compromise the detoxification ability of bats, as well as their excretion capacity. Other authors suggested Ni > Cd > Cr > Pb for the male reproductive toxicity order in mice.^21^ Here, for bats, we are proposing Ni > Pb > Cd > Cr male reproductive toxicity. Studies in rats showed that the other metals (Ni, Pb, and Cd) also induced reproductive toxicity at higher concentrations and/or exposure times.^10,50,51^

### Study Limitations

Using wild bats as models, although bringing species-specific information needed for ecotoxicological studies, imposes certain limitations inherent in the lack of knowledge about the individuals prior to capture. In order to minimize potential variation, we selected only adult males from the same forest fragment, in the same season and year, and ran an acclimatation period of 4 days before the experiments, when physiological characteristics such as food and water intake, flight, and other behaviors are observed. No animal showed any impairment in food consumption, water intake, or flight ability during the experiment. Our study suggests that bats may be more sensitive to metals and that, for the reproductive system, the order of toxicity in bats may differ from that observed in mice. For a better comparison with murine models and more robust conclusions, further studies on bats should be conducted and compiled for a more comprehensive assessment.

## Conclusions

Taken together, our results draw attention to oxidative and tissue damage from heavy metal exposure in fruit bats, even under a short exposure time. Our results indicate that bats may be susceptible to the effects of heavy metals, and the liver seems to be a more sensitive tissue, due to the severe histological damage, including necrosis and lipidic areas, found in this tissue. In addition, we propose the following order of metal toxicity, based on the degree of oxidative and histological damage: Ni > Pb > Cr = Cd. Ni-exposed animals showed the highest IBR in the liver and testes, where they showed oxidative damage and also showed histological damage in the kidneys and necrosis areas in the liver. Considering that, the most worrisome metal is Ni and must be a priority in pollutant mitigation plans, such as bioremediation. Also, findings of higher levels of protein carbonyl and vacuolization in testes demonstrate that Cr, constantly released into the environment, may affect the reproductive capacity of bats and therefore their ecological contribution. More studies with different concentrations and exposure times are needed to better assess other toxicological effects. Assessing and monitoring bat populations in highly contaminated areas is critical to better understand the damage caused by environmental pollution to key ecological species.

## Supplementary Material



## Notes

Star plot of tissues exposed to heavy metals; total area of the star plots representing the value of the IBR index (PDF)
