# Supplementary material for: Individual Acute Exposures to Low Concentrations of Cadmium, Chromium, Lead, and Nickel Affect Oxidative Stress and Pathological Markers in Fruit Bats
Source: ACS Omega. 2025 May 8;10(19):19797–807. doi: 10.1021/acsomega.5c00991 (PMC12096224; doi:10.1021/acsomega.5c00991)
Supplement: Supplementary file 1 [file ao5c00991_si_001.pdf]

Individual acute exposures to low concentrations of cadmium, chromium, lead, and nickel affect oxidative stress and pathological markers in fruit bats

Ana Luiza Fonseca Destro*[a], Thaís Silva Alves[a], Fernanda Ribeiro Dias[b], Reggiani Vilela Gonçalves[a], Jerusa Maria de Oliveira[d], Leandro Licursi de Oliveira[c], Mariella Bontempo Freitas[a]

[a] Federal University of Viçosa, Department of Animal Biology, 36570-900, Viçosa, MG, Brazil.
[b] Federal University of Triângulo Mineiro, Department of Structural Biology, 38025-350, Uberaba, MG, Brazil.
[c] Federal University of Viçosa, Department of General Biology, 36570-900, Viçosa, MG, Brazil.
[d] Federal University of Alagoas, Physics Institute,57072450, Maceió, AL, Brazil.

Corresponding author:
*Ana Luiza Fonseca Destro, Department of Animal Biology, Federal University of Viçosa, Viçosa Minas Gerais, 36570-900, Brazil.
Email: nina.destro@gmail.com

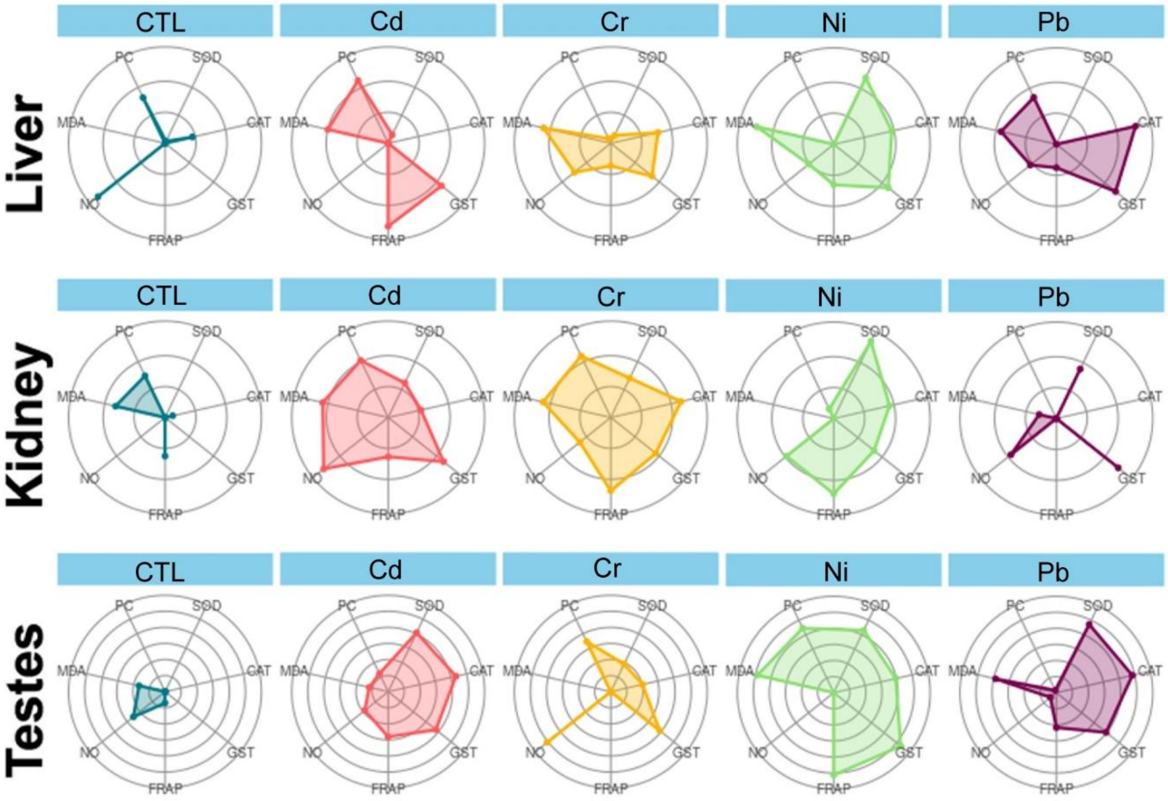

Fig 1S. Star plot of tissues exposed to heavy metals. The total area of star plots represents the value of the IBR index.
